# Synthesis and Odor Evaluation of Five New Sulfur-Containing Ester Flavor Compounds from 4-Ethyloctanoic Acid

**DOI:** 10.3390/molecules15085104

**Published:** 2010-07-29

**Authors:** Yuping Liu, Haitao Chen, Decai Yin, Baoguo Sun

**Affiliations:** 1 College of Chemistry and Chemical Engineering, Shaanxi University of Science and Technology, Xi’an 710021, China; 2 School of Chemical and Environmental Engineering, Beijing Technology and Business University, Beijing 100048, China

**Keywords:** sulfur-containing compounds, flavor, synthesis, odor characteristic

## Abstract

Five sulfur-containing flavor compounds were synthesized for the first time by the reaction of 4-ethyloctanoyl chloride with sulfur-containing alcohols or mercaptans. The synthesized compounds are 3-(methylthio)propyl 4-ethyloctanoate, 2-methyl-3-tetrahydro-furanthiol 4-ethyloctanoate, 4-methyl-5-thiazoleethanol 4-ethyloctanoate, 2-furan-methanethiol 4-ethyloctanoate and 2-methyl-3-furanthiol 4-ethyloctanoate. These five synthetic sulfur-containing ester flavor compounds all have meaty odor and might be used in foods if approved for this purpose in the future.

## 1. Introduction

Esters are important flavor compounds because of the large number of accessible ester compounds, their occurrence in a wide range of natural sources, various odor characteristics and their wide range of uses in flavourings. The odor of esters is related to the organic acid and alcohol from which they derived. Moreover, the odor intensity of esters decreases with the increase of molecular weight; however, the flavor industry needs esters with strong odors and high molecular weight to prolong the duration of the odor of flavorings. Esters containing sulfur can be synthesized to yield flavor substances with strong odor and high molecular weight, as flavor compounds containing sulfur exhibit very low odor threshold levels and very good odor characteristics.

The sulfur-containing esters can be divided into two classes; one in which the sulfur atom belongs to the organic acid, such as CH_3_SCH_2_CH_2_COOCH_3_, and the other in which the sulfur atom is from an alcohol or mercaptan (AOM), such as CH_3_COOCH_2_CH_2_SCH_3_ or CH_3_COSCH_2_CH_2_CH_3_. The first sulfur-containing ester class can be synthesized by the thia-Michael addition reaction, which is facile and efficient [1,2,3]. The second ester class can be synthesized by several methods. One method is the preparation of the ester from AOM and an organic acid in the presence of a catalyst, which can be basic, like triethylamine [4] or acidic [5,6] like solid superacids or *p*-toluenesulfonic acid. A second method is treatment of AOM with an anhydride yielding the corresponding ester [6,7,8,9]; mercaptans can be used directly as the starting reagent or can be obtained by reducing an alkyl disulfide with tributyl phophine [9]. A third method is the preparation of the esters by reaction of an acyl chloride with AOM [10,11,12,13,14,15]; however, the hydrogen chloride side product must sometimes be eliminated by pyridine addition. The fourth method is the synthesis of esters by reaction of a haloalkane with the salt of a thio-organic acid in THF [16]; these starting materials are not readily available, so this method is mainly used in the synthesis of esters from thioacetic acid. 

**Scheme 1:**
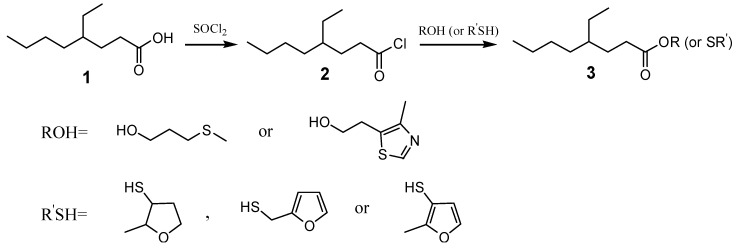
Reaction route.

**Figure 1:**
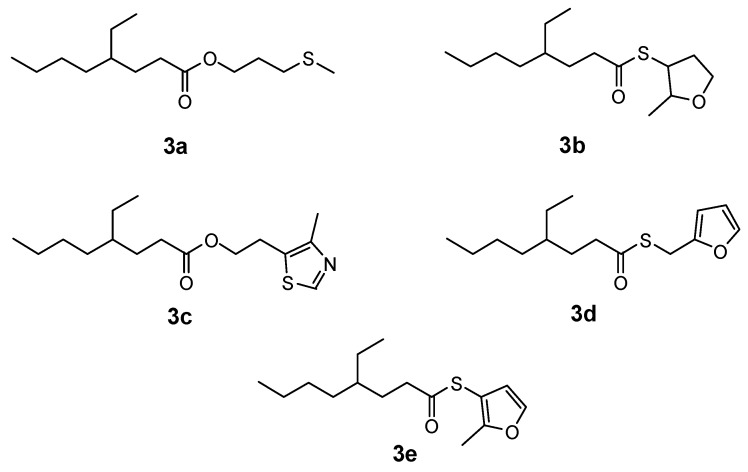
Structures of compounds **3a-e**.

4-Ethyloctanoic acid has a low odor threshold of 1.8 ppb [17] and possesses an odor variously described as waxy, fatty, creamy, moldy and cheesy, with animal-like nuances; it is used in flavoring for goat meat with fatty, meaty nuances and savory notes [18]. 3-(Methylthio)propanol, 4-methyl-5-thiazole ethanol, 2-methyl-3-furanthiol, 2-methyl-3-tetrahydrofuranthiol and 2-furanmethanethiol are all very important sulfur-containing flavor compounds in formulating meaty flavorings [19,20,21,22] and are also used to synthesize acetates. Their corresponding 4-ethyloctanoates would have desirable high molecular weights but have never been synthesized. We have now synthesized and evaluated the odors of the esters derived from 4-etyloctanoic acid with the above AOMs. The carbonyl in 4-ethyloctanoic acid is a weak electrophile, so 4-ethyloctanoyl chloride is synthesized as an intermediate to increase the electrophilic properties of the carbonyl group. 4-Ethyloctanoyl chloride (**2**) was synthesized by the reaction of thionyl chloride with 4-ethyloctanoic acid (**1**), and then **2** reacted with either 3-(methylthio)propanol, 2-methyl-3-tetrahydrofuranthiol, 4-methyl-5-thiazoleethanol, 2-furanmethane-thiol and 2-methyl-3-furanthiol, respectively (Scheme 1), to give their corresponding esters **3a**-**3e **(Figure 1). 

The esters underwent odor evaluation, which demonstrated that these five synthetic sulfur-containing flavor compounds all have meaty odor and might be used in foods if approved for this purpose in the future.

## 2. Results and Discussion

The effects of different mole ratios of materials on the yield were investigated using three different ratios under the same experimental conditions, and the results are shown in Table 1. Because the 4-ethyloctanoic acid used was a racemic compound, the five synthesized flavor compounds are also racemic.

**Table 1:** Effect of different mole ratio of materials on the yield.

Compound	Yield (%)		
	*n*_ (alcohol or mercaptan) _: *n*_ (4-ethyloctanoyl chloride)_		
	1.2:1	1.6:1	2.0: 1
**3a**	85%	90%	94%
**3b**	67%	78%	94%
**3c**	93%	89%	87%
**3d**	93%	94%	95%
**3e**	79%	84%	86%

The yields of **3a**, **3b**, **3d** and **3e** increased as the ratio of mercaptan (or alcohol) to 4-ethyloctanoyl chloride increased, while the yield of **3c** decreased as the ratio of alcohol to 4-ethyloctanoyl chloride increased. This phenomenon was related to the characteristics of the starting materials involved in each reaction. In the reaction producing **3c**, the 4-methyl-5-thiazoleethanol used as a starting material has multiple nucleophilic centers (O, S and N), which may react with 4-ethyloctanoic chloride to give the product or byproducts. Although the reaction temperature and steric-hindrance effect were not appropriate to form byproducts, the yield increased as the ratio of 4-methyl-5-thiazoleethanol to 4-ethyloctanoyl chloride increased. This may account for the low yield of **3c**. 

The hydrogen chloride generated in the reactions to produce **3d** and **3e** induces the breakage of furan ring structure found in the starting material and the formation of resin-like byproducts. Pyridine was used in these reactions to trap the hydrogen chloride [23]. Pyridine can also react with 4-ethyl-octanoyl chloride to give pyridine 4-ethyloctanoate, which readily forms the desired ester product with the mercaptan [24].

2-Methyl-3-furanthiol and 2-furanmethanethiol are isomers, where the latter one is more reactive than the former. The mercapto group of 2-methyl-3-furanthiol is adjacent to the conjugated system, which weakens its nucleophilicity. Consequently, the yield of **3e** is lower than that of **3d**.

The odor evaluation results are shown in Table 2. The results indicate that the five synthetic sulfur-containing flavor compounds all have meaty odor, especially **3b**, **3d** and **3e**. Amongst those five compounds, the three derived from mercaptan and 4-ethyloctanyl chloride (**3b**, **3d** and **3e**) have stronger odors than the two derived from a sulfur-containing alcohol and 4-ethyloctanyl chloride (**3a** and **3c**). All five flavor compounds have relatively high molecular weights (> 260) and are therefore less volatile, which can prolong the flavoring lifetime to improve the flavoring quality. 

**Table 2:** The results of odor evaluation.

Compound	Odor characteristics
**3a**	meaty, sauce, savory odor with a slight potato, radish and sulfury nuance
**3b**	meaty, onion, metallic, sulfury odor with a slight hot nuance
**3c**	a slight meaty, roasted and, burnt odor with sesame oil and fatty nuance
**3d**	meaty, roasted meat, onion, sulfury odor with a slight garlic nuance
**3e**	meaty, brothy, sulfury roasted odor with a slight sweet note

## 3. Experimental

### 3.1. General

The samples were analyzed by Varian CP3800 gas chromatography. Mass spectra were obtained using a Waters Micromass Q-TOF mass spectrometer. ^1^H-NMR and ^1^^3^C-NMR spectra were obtained on a Bruker DRX-300 nuclear magnetic resonance spectrometer. IR spectra were acquired on a Nicolet Avater 370 Fourier transform infrared spectrometer. Odor evaluation was carried out by gas chromatography-olfactometry.

### 3.2. Synthesis of 4-ethyloctanoyl chloride (**2**)

To 4-ethyloctanoic acid (0.2 mol) was added thionyl chloride (0.28 mol) at 30 ºC. The resulting solution was refluxed for 6 hours at 80 ºC, and the excess thionyl chloride was evaporated *in vacuo* to collect the distillate at 74 ºC/0.4 kPa and give the 4-ethyloctanoyl chloride, yield 97%. ^1^H-NMR (CDCl_3_, *δ* ppm) 2.75-2.80 (t, 2H), 1.58-1.60 (m, 2H), 1.16-1.22 (m, 9H), 0.77-0.83 (m, 6H); ^13^C-NMR (CDCl_3_, *δ* ppm) 174.10 (C=O), 44.81 (CH_2_), 37.89 (CH), 32.31 (CH_2_), 28.67 (CH_2_), 28.25 (CH_2_), 25.41 (CH_2_), 22.98 (CH_2_), 14.06 (CH_3_), 10.60 (CH_3_); IR (KBr,cm^-1^) 2960, 2929, 2860, 1801, 1458, 1404, 1380, 956, 709, 686, 596. 

### 3.3. General Synthesis of Compounds **3a-c**

To alcohol (or mercaptan) (0.1 mol) was added 4-ethyloctanoyl chloride (0.05 mol) at 30 ºC, and the resulting solution was refluxed for 4 hours at 70 ºC. After cooling, ethyl ether (30 mL) was added. The mixture was washed with 10% Na_2_CO_3_ aqueous solution and saturated NaCl aqueous solution to pH 6-7, then the sample was dried with anhydrous NaSO_4_ and filtered. The ethyl ether was evaporated *in vacuo* and the mixture was weighed. The composition of the product mixture was determined by gas-chromatography, and the yield was calculated. The product was further purified using distillation under the reduced pressure. 

*3-(Methylthio)propyl 4-ethyloctanoate* (**3a**). Yield 94%; b.p. 142 ºC/0.4 kPa; refractive index (n_D_^20^) 1.4654; MS (EI), m/z, 260.1812 (M^+^), formula C_14_H_28_O_2_S, (calc. mass 260.1810). ^1^H-NMR (CDCl_3_, *δ* ppm) 4.14-4.18 (t, 2H), 2.53-2.58 (t, 2H), 2.25-2.30 (t, 2H), 2.10 (s, 3H), 1.87-1.96 (m,2H), 1.55-1.60 (m, 2H), 1.23-1.30 (m, 9H), 0.84-0.90 (m, 6H); ^13^C-NMR (CDCl_3_, *δ* ppm) 173.82 (C=O), 62.65 (OCH_2_), 38.25 (CH), 32.33 (CH_2_), 31.60 (CH_2_), 30.50 (CH_2_), 28.66 (CH_2_), 28.15 (CH_2_), 28.12 (CH_2_), 25.42 (CH_2_), 22.91 (CH_2_), 15.30 (SCH_3_), 13.94 (CH_3_), 10.57 (CH_3_); IR (KBr,cm^-1^) 2958, 2925, 2864, 1737, 1460, 1383, 1349,1241, 1168, 1108, 1024, 960. 

*2-Methyl-3-tetrahydrofuranthiol 4-ethyloctanoate *(**3b**). Yield 94%; b.p. 140 ºC/0.2 kPa; refractive index (n_D_^20^) 1.4830; MS (EI), m/z, (M^+^) 272.1813, formula C_15_H_28_O_2_S, (calc. mass 272.1810). ^1^H- NMR (CDCl_3_, *δ* ppm) 4.09-4.13 (m, 0.5H), 3.95-4.03 (m, 0.5H), 3.89-3.93 (m, 1H), 3.70-3.82 (m, 1.5H), 3.48-3.53 (m, 0.5H), 2.41-2.53 (m,3H), 1.82-1.86 (m, 1H), 1.59-1.61(m, 2H), 1.16-1.26 (m, 12H), 0.80-0.88 (m, 6H); ^13^C-NMR (CDCl_3_, *δ* ppm) 199.48 (C=O), 80.19 (CH-O), 66.81 (CH_2_-O), 46.47 (CH-S), 41.68 (CH_2_), 38.33 (CH), 33.39 (CH_2_), 32.51 (CH_2_), 29.05 (CH_2_), 28.83 (CH_2_), 25.60 (CH_2_), 23.11(CH_2_), 17.00 (CH_3_), 14.19 (CH_3_),.10.77 (CH_3_); IR (KBr,cm^-1^) 2959, 2928, 2872, 1692, 1456, 1380, 1352, 1315, 1188, 1111, 1063, 1022, 990, 858. 

*4-Methyl-5-thiazoleethanol 4-ethyloctanoate *(**3c**). Yield 93%; b.p. 175 ºC/0.4 kPa; refractive index (n_D_^20^) 1.4920; MS (EI), m/z, 297.1766 (M^+^), formula C_16_H_27_O_2_SN, (calc. mass 297.1763); ^1^H-NMR (CDCl_3_, *δ* ppm) 8.55 (s, 1H), 4.17-4.21 (t, 2H), 3.03-3.08 (t, 2H), 2.38 (s, 3H), 2.22-2.27 (m,2H), 1.53-1.55 (m, 2H), 1.19-1.24 (m, 9H), 0.78-0.87 (m, 6H); ^13^C-NMR (CDCl_3_, *δ* ppm) 173.90 (C=O), 149.86 (S-CH=N), 149.77 (N-C), 126.75 (S-C), 63.81 (O-CH_2_), 38.28 (CH), 32.36 (CH_2_), 31.65 (CH_2_), 28.72 (CH_2_), 28.07 (CH_2_), 25.77 (CH_2_), 25.45 (CH_2_), 23.03 (CH_2_), 14.90 (CH_3_), 14.10 (CH_3_), 10.68 (CH_3_); IR (KBr,cm^-1^) 2958, 2926, 2860, 1738, 1543, 1458, 1416, 1378, 1238, 1167, 1106, 1035, 915, 847, 786. 

### 3.4. General Synthesis of Compound **3d-e**

A mixture of 2-furanmethanethiol(or 2-methyl-3-furanthiol) (0.1 mol), pyridine (0.1 mol) and dichloromethane (40 mL) was prepared and to this was added 4-ethyloctanoyl chloride (0.05 mol) at 30 ºC. The resulting solution was refluxed for 6 hours at 45 ºC. At the end of this period, the mixture was filtered to remove solids, and the filtrate was washed with saturated NaCl aqueous solution. The organic phase was dried with anhydrous NaSO_4_ and filtered. Dichloromethane and pyridine were evaporated *in vacuo* and the mixture weighed. The composition of the product was determined by gas-chromatography and the yield calculated. The product was further purified via distillation under reduced pressure. 

*2-Furanmethanethiol 4-ethyloctanoate* (**3d**). Yield 95%; b.p. 142 ºC/0.3 kPa; refractive index (n_D_^20^) 1.5005; MS (EI), m/z, 268.1500 (M^+^), formula C_15_H_24_O_2_S, (calc. mass 268.1497); ^1^H-NMR (CDCl_3_, *δ* ppm) 7.31 (m, 1H), 6.27 (m, 1H), 6.19-6.20 (m, 1H), 3.03-3.08 (t, 2H), 4.13 (s, 2H), 2.51-2.56 (m, 2H), 1.63 (m, 2H), 1.23-1.28 (m, 9H), 0.81-0.90 (m, 6H); ^13^C-NMR (CDCl_3_, *δ* ppm) 198.54 (C=O), 150.61 (=C-O), 142.13 (=CH-O), 110.53 (CH=), 107.82 (CH=), 41.41 (CH_2_), 38.24 (CH), 32.42 (CH_2_), 28.73 (CH_2_), 25.63 (CH_2_), 25.55 (CH_2_), 25.51 (CH_2_), 23.03 (CH_2_), 14.10 (CH_3_), 10.68 (CH_3_); IR (KBr, cm^-1^) 2958, 2927, 2859, 1694, 1596, 1502, 1460, 1380, 1246, 1151, 1071, 1010, 935, 885, 807, 735, 597. 

*2-Methyl-3-furanthiol 4-ethyloctanoate* (**3e**). Yield 86%; b.p. 134 ºC/0.2 kPa; Refractive index (n_D_^20^) 1.4960; MS (EI), m/z, 268.1499 (M^+^), formula C_15_H_24_O_2_S, (calc. mass 268.1497); ^1^H-NMR (CDCl_3_, *δ* ppm) 7.35-7.36 (d, 1H), 6.31-6.32 (d, 1H), 2.59-2.62 (t, 2H), 2.26 (s,3H), 1.63-1.70 (m, 2H), 1.26-1.32 (m, 9H), 0.84-0.92 (m, 6H); ^13^C-NMR (CDCl_3_, *δ* ppm) 197.49 (C=O), 155.92 (=C-O), 140.88 (=CH‑O), 114.78 (CH=), 104.30 (S-C), 40.70 (CH_2_), 38.25 (CH), 32.41 (CH_2_), 28.71 (CH_2_), 28.68 (CH_2_), 25.50 (CH_2_), 22.95 (CH_2_), 13.99 (CH_3_), 11.78 (CH_3_), 10.62 (CH_3_); IR (KBr, cm^-1^) 2959, 2926, 2859, 1715, 1588, 1514, 1387, 1228, 1129, 1090, 1020, 940, 889, 728, 648, 603, 525. 

### 3.5. Odor evaluation

These compounds were purified by column chromatography. Odor assessments were carried out by using a Agilent 6890 GC coupled with a Sniffer 9000 system. Separations were achieved with a HP-5 capillary column, 30 m × 0.250 mm; with a programmed temperature ramp, (200 ºC, holding for 2 min, then programmed to 300 ºC at 10 ºC/min and held at 300 ºC for 2 min; injection temperature, 280 ºC) detector (FID) temperature at 300 ºC. By one ‘Y’ shape glass splitter, the column effluent was divided (1:1) between the flame ionization detector (FID) and the olfactometer (Sniffer 9000). The effluent to the odor port was enclosed with a stream of humidified air of 6 mL/min and transferred to the glass detection cone by one long capillary at the temperature of 280 ºC. Five trained assessors were selected for smelling and recording the odor characteristics [25,26].

## 4. Conclusions

We report the synthesis and odor evaluation of sulfur-containing esters derived from 4-ethyl-octanoic acid. The structures of five new flavor compounds were characterized by MS, ^1^H-NMR, ^13^C-NMR and IR, and odor evaluation indicated they all have meaty odors. This research will be helpful to develop new flavor compounds based on organic acid and sulfur-containing alcohols (or mercaptans). 
